# Segregation of the Tuberculous

**Published:** 1912-07

**Authors:** 


### Segregation of the Tuberculous

DR. HARVEY D. WILEY has suggested, how seriously we do not know, that all tuberculous persons should be isolated on some island. His suggestion is criticized as not in accord with the principles of humanity. Having personally made practically the same suggestion, quite a number of years ago, and probably with the same realization as Dr. Wiley, that it is not feasible at present, it may not be out of place to raise some questions as to its merits and demerits.

As to the humanity of the proposition, we can appreciate fully that the removal of a tuberculous patient from his home may occasion considerable suffering. For this suffering, we have every sympathy; nostalgia is no laughing matter. On the other hand, it must be conceded that there is no more essential cruelty in isolating a tuberculous than leprous patient and no more in sending a consumptive from home as a matter of prophylaxis than of therapeutics. It is not to be supposed that the isolation of all tuberculous patients would be done with brutal disregard for their comfort, any more than that this should be the case for some consumptives. One the contrary, compulsory, general quarantine would, as a matter of common sense and common humanity, imply a careful assortment of cases according to location, stage and other features of the disease, the most expert attention to therapeutic indications and the utmost pains to alleviate the suffering of hopeless cases.

Those who condemn isolation on humanitarian grounds forget one very important fact. Under present methods, a great many patients receive practically no care at all until confined to bed in the last stage of the disease; there is no efficient means to discipline them so as to afford a reasonable chance of cure; on account of poverty, many lack scientific treatment and even good nursing, however much they may enjoy the love and companionship of their homes; even from the sentimental standpoint, dying at home is balanced by many apprehensions as to the safety and future welfare of the family. Imagine, on the other hand, that compulsory isolation of the tuberculous were a fact. It would obviously operate as early as a case could be detected and the self interest of the community would render concealment of cases very difficult. The present mortality, is probably 90%. In carefully managed series of early cases, it is somewhere about 50%, certainly not higher than 75% and according to some reports, as low as 25%. There is no reason why, allowing for the first embarrassment of providing accommodations and care for a sudden large influx of patients, the same saving of life could not be effected for patients sent to suitable places as a matter of safety for the community as for those sent, under present conditions, for their own sake. The enormous aggregate saving of life, preservation of families, and restoration to more or less robust health strikes us as being essentially humane, even if contrary to the preferences of the patients. Critics on the humanitarian side forget that, after the first year or so of the operation of a compulsory isolation law, it would apply only to a very small minority merely sent away to die in the interests of survivors. The great mass of those affected by such a law, after its inception, would have an exceedingly good chance of recovery.

Let us consider the proposition now, solely as a matter of prophylaxis. The great majority of all cases of tuberculosis in man are due to the human variety of the bacillus. Probably not more than 5 or 10% are due to the bovine type and the risk of infection of human beings from bovine sources has been shown to be comparatively slight, though not nil, as was at first claimed by Koch or, at least, by his interpreters. There are certain cases in the lower animals due to the human variety of the bacillus but these occur mainly in domestic animals closely associated with human patients and the prophylaxis depends mainly upon the disposition of the human case. In other words, the great majority of human cases of tuberculosis, are due to previous human cases.

The tubercle bacillus, though wide spread, is a comparatively feeble organism, killed by sunlight in a few hours, killed by diffuse daylight within a few weeks, fairly amenable to control by cleansing and disinfectant treatment of fomites. The great majority of persons are fairly resistant to it in a state of ordinary health, provided the infection is not massive. In other words, if the human sources of infection could be removed, opportunities for further infection could, for the most part, be quickly removed and such cases as did occur from fomites overlooked, would decrease in geometric progression.

A very important detail is that, with the tuberculous patient out of the way, not only would disinfection be much more feasible but persons still exposed to persisting fomites would be enabled to avoid the fatigue, confinement and reiterated dangers of implantation of bacilli, which now lead to the development of cases in the wake of another.

The problem is further simplified by the fact, or at least, apparent demonstration by experience that the "striking distance" of the tubercle bacillus is not great. That is to say, the suggestion of an "island" for the isolation of the tuberculous, is merely a figure of speech. There is no need of calculating the size of an island at an enormous distance from the normal population, where all tuberculous patients may be isolated. The problem may be reduced to convenient units of population and suitable locations are available, at no prohibitive expense, and within at most a night's ride of practically any point in the country. While isolation should be isolate, it seems to be true that no tremendous distance of water or land, need intervene between a colony of tuberculous patients and ordinary settlements. With such reasonable care as to wind-borne and water-borne infection as can easily be carried out in a colony, there seems to be no appreciable danger even to those in close proximity. Moreover, with strict precautions, even the attendants run little risk and isolation need not necessarily preclude brief visits from friends.

The more we think about the proposition, the more it impresses us as worthy of practical consideration. We should be glad of an expression of opinion from our readers.

